# Factors Associated with Poor Glycemic and Lipid Levels in Ambulatory Diabetes Mellitus Type 2 Patients in Asmara, Eritrea: A Cross-Sectional Study

**DOI:** 10.1155/2020/5901569

**Published:** 2020-01-28

**Authors:** Oliver Okoth Achila, Millen Ghebretinsae, Abraham Kidane, Michael Simon, Shewit Makonen, Yohannes Rezene

**Affiliations:** ^1^Department of Clinical Laboratory Sciences, Orotta College of Medicine and Health Sciences, Eritrea; ^2^Department of Clinical Laboratory Services, Asmara College of Health Sciences (ACHS), Eritrea

## Abstract

**Objective:**

There is a dearth of relevant research on the rapidly evolving epidemic of diabetes mellitus (particularly Type 2 diabetes mellitus) in sub-Saharan Africa. To address some of these issues in the Eritrean context, we conducted a cross-sectional study on glycemic and lipid profiles and associated risk factors.

**Methods:**

A total of 309 patients with diabetes mellitus on regular follow-up at the Diabetic and Hypertensive Department at Halibet Regional Referral Hospital, Asmara, were enrolled for the study. Data on specific clinical chemistry and anthropomorphic parameters was collected. Chi-squared (*χ*^2^) test or Fischer's exact test was used to evaluate the relationship between specific variables. Multivariate logistic regression (backward: conditional) was undertaken to identify the factors associated with increased odds of suboptimal values in glucose and specific lipid panel subfractions.

**Results:**

High proportions of patients (76.7%) had suboptimal levels of HbA1c with a mean ± SD of 8.6% ± 1.36, respectively. In multivariate regression analysis, the likelihood of HbA1c ≥ 7% was higher in patients with abnormal WHR (AOR = 3.01, 95% CI, 3.01 (1.15–7.92 = 0.024)) and in patients without hypertension (AOR = 1.97, 95% CI (1.06–3.56), *p* = 0.021). A unit reduction in eGFR was also associated with HbA1c ≥ 7% (AOR = 0.99, 95% CI (0.98–1 = 0.031)). In a separate analysis, the data shows that 80.9% of the patients had dyslipidemia. In particular, 62.1% of the patients had TC ≥ 200 mg/dL (risk factors: sex, hypertension, and HbA1c concentration), 81.6% had LDL‐C ≥ 100 mg/dL (risk factors: sex and hypertension), 56.3% had TG ≥ 150 (risk factors: sex, HbA1c, and waist circumference), 62.8% had abnormal HDL-C (risk factors: waist circumference), 78.3% had non‐HDL < 130 mg/dL (risk factors: duration of disease, reduced estimated glomerular filtration rate, and HbA1c), and 45.3% had abnormal TG/HDL (risk factors: sex, age of patient, FPG, and waist circumference).

**Conclusions:**

The quality of care, as measured by glycemic and specific lipid targets, in this setting is suboptimal. Therefore, there is an urgent need for simultaneous improvements in both indicators. This will require evidence-based optimization of pharmacological and lifestyle interventions. Therefore, additional studies, preferably longitudinal studies with long follow-up, are required on multiple aspects of DM.

## 1. Introduction

Diabetes mellitus (DM) describes a constellation of metabolic disorders characterised by hyperglycemia and disturbances to carbohydrate, fat, and protein metabolism resulting from insulin deficiency, insulin resistance, or both [1]. Type 2 diabetes mellitus (T2DM), a heterogeneous, complex, multifactorial metabolic disorder characterised by chronic hyperglycemia, is the most predominant form [2]. Leveraging the highest quality epidemiological data, the International Diabetes Federation (IDF) estimated that, globally, there were 463 million people (uncertainty interval: 368-600.6 million) living with T2DM in 2019 [2]. The figure is projected to rise to 700.2 million (uncertainty interval: 540.7-904.6) by 2045. Glaring disparities in projected regional increases in DM were also reported with sub-Saharan Africa (SSA) expected to register the steepest regional growth rates (252.7%) by 2045. Importantly, the increasing burden of DM is not accompanied by substantial improvements in the control and/or management of infectious diseases or undernutrition.

A fundamental implication of the rapid increase in T2DM in SSA is a substantial increase in DM-associated mortality and morbidity [2–4]. The mortality and morbidity, driven in part by suboptimal management of the disease, are mediated by an array of macrovascular (coronary, cerebrovascular, and peripheral vascular diseases) and microvascular (retinopathy, neuropathy, and nephropathy) complications [1, 5]. Other hitherto underappreciated complications include infections, mental illness, cognitive decline, lung diseases, and some cancers [5]. Multiple pathophysiological processes underlying these complications have been proposed [6]. However, dyslipidemia is currently regarded as one of the most important mediators of macrovascular disease (e.g., atherosclerotic vascular diseases (ASVD)) in patients with DM [6–8].

Despite the growing burden of DM [9] and the existence of a fairly robust clinical armamentarium (pharmacological and nonpharmacological) for optimal management of the disease [10], several studies have demonstrated that the quality of care for patients, particularly in SSA, is generally wanting [11–13]. In particular, substantial variability within and between countries in the magnitude of poor glycemic control has also been reported [12, 13]. This contextual uniqueness has been associated with inter- and intracountry variation in multiple patient-centred, hospital-based, and healthcare system factors. Prominent factors include ethnicity, high levels of poverty, limited in-country funding for DM clinics, cultural obstacles, underdiagnosis, poor laboratory infrastructure, uncoordinated care, and shortage of physicians or other medical health specialists [14]. Emphasising these facts, some scholars have stressed the fact that very few countries in the continent can afford to broadly screen and treat the various complications associated with DM [3]. More importantly, there is a paucity of published information on the quality of care for patients with DM.

In Eritrea, unpublished data from the country's health management system indicates that noncommunicable diseases (NCDs) like DM are increasingly becoming a major public health concern surpassing several infectious diseases (e.g., malaria, tuberculosis, and human immunodeficiency virus (HIV)) in terms of all-cause morbidity and mortality [15]. Indeed, a recent publication reported a high incidence of DM-associated amputations and ASCVD in the country [15]. Despite the growing concern, we could not locate any recently published information on glycemic control or other aspects of DM management. Existing literature is dated or limited in scope [16]. Moreover, and given the lack of longitudinal studies in this setting, periodical cross-sectional studies may help in highlighting improvements, deteriorations, or sustained optimal DM care. To address some of these issues, we conducted a cross-sectional study on glycemic control and lipid profiles and associated risk factors in one of the major DM follow-up clinics in Asmara, Eritrea.

## 2. Participants and Methods

### 2.1. Study Setting and Period

This study was conducted at Halibet Regional Referral Hospital, Zoba Maekel, Asmara, Eritrea, from February 2017 to May 2017. Established in 1992 primarily as a regional hospital, the hospital was subsequently upgraded to a tertiary level facility in 2010. It provides medical services to the approximately 560 000 residents of Asmara and adjoining catchment areas. Halibet Regional Referral Hospital and Haz-Haz Hospital are the only facilities within Asmara with DM follow-up and care units and are participants in Eritrean government universal health insurance. The diabetes clinic provides regular monitoring of DM patients where they are attended by a multidisciplinary team of internists, medical residents, pharmacists, and general nurses during scheduled follow-up visits. The patients are also provided with medication after paying a small nominal fee.

### 2.2. Study Design and Data Collection

The study was a descriptive cross-sectional evaluation of DM patients attending a follow up facility. Variables abstracted from patient charts included age, disease (DM) duration, hypertension, and other DM-related comorbidities. A questionnaire, translated to Tigrigna (a common local dialect) and incorporating queries on a range of well-established lifestyle-related DM risk factors, was employed to collect information on demographic data (level of education, occupation), lifestyle habits (exercising, smoking, and frequency of alcohol intake), and family history of DM (individuals with a first-degree family member with a diagnosis of DM made by a qualified physician).

Clinical laboratory data, including hemoglobin A1c (HbA1c), fasting plasma glucose (FPG), creatinine (Cre), C-reactive protein, total cholesterol (TC), low-density lipoprotein cholesterol (LDL-C), high-density lipoprotein cholesterol (HDL-C), and triglycerides (TG), were obtained from subsequent laboratory analysis of the sampled blood specimen. Fasting plasma glucose (FPG) from two consecutive visits prior to the present specimen collection date was also retrieved from the patient's records.

### 2.3. Sample Size Calculation and Participant Recruitment and Selection

The sample size was estimated using the Cochran formula. The total sample size (*n* = 288) was subsequently adjusted by a factor of 10% to give a sample size of 316. The DM clinic record book was used as a sampling frame for random selection of the eligible study participant. A systematic sampling procedure, involving the selection of every 2^nd^ person who visited the diabetes clinic during the study period, was undertaken. Inclusion criteria included Type 2 DM (diagnosis was based on patient records) patients currently being treated with daily medication and individuals who were willing to grant consent. Exclusion criteria were based on the following consideration: hospitalized and/or individuals with cognitive impairment (dementia or other psychiatric disorders), patients with overt thyroid dysfunction, and individuals who were unwilling to grant consent.

### 2.4. Ethical Approval and Consent to Participate

Ethical approval for the study and experimental protocols used was obtained from the Eritrean Ministry of Health (MOH) research ethical committee and Asmara College of Health Sciences (ACHS) Scientific and Ethical Eommittee. Informed consent was obtained from all participants after extensive explanation of the study objective or purpose, study procedures, and possible adverse effects. All participants were duly informed of their rights to refuse or terminate their participation in the study. Information on the maintenance of data confidentiality and integrity was also provided. Strict adherence to approved laboratory protocols and GCP was observed during specimen collection, processing, and testing.

### 2.5. Measures and Operational Definitions

Independent variables and outcome measures were defined as follows.

#### 2.5.1. Lifestyle/Behavioural Factors

Lifestyle factors including alcohol consumption and cigarette/tobacco smoking were defined as follows. Smokers were categorized as (a) nonsmokers (individuals who had not smoked or previous smokers who had quit for more than one year prior to the study commencement) and (b) smokers (current user of at least one cigarette a day or occasional smokers) [17]. Further, self-reported consumption of any alcoholic brew, at least twice weekly, was logged as evidence of alcohol consumption [17]. Participants were also required to indicate whether they were adhering to prescribed clinical guidelines on dieting or physical activity. Responses were classified on a yes or no schema.

#### 2.5.2. Anthropometric

Anthropometric data such as weight, height, body mass index (BMI), and waist and hip circumference were measured. Weight was measured using a calibrated medical weight balancing scale (Zhongshan Camry Electronic Co. Ltd., China) secured on a firm level surface and was reported in kilograms (kg). Waist circumference (cm) was measured with a tape as the point between the iliac crest and the costal margin in the midaxillary line while the patient was standing and breathing normally. The widest portion of the buttocks was taken as the hip circumference (cm). The waist-to-hip ratio (WHR) (a measure of abdominal obesity) was calculated as waist (cm)/hip (cm). Abnormal WHR was defined as a ratio > 0.90 for men and >0.80 for females. Further, BMI was defined as weight (kg)/(height (m))^2^. Correspondingly, being overweight was defined as having a BMI ≥ 25.0‐29.9 kg/m^2^, and obesity as having a BMI ≥ 30 kg/m^2^ as per World Health Organization (WHO) criteria [18]. DM duration was established by subtracting present age from age at diagnosis (a value obtained from the patient's hospital records).

#### 2.5.3. Glycemic Control

Poor glycemic control was defined as HbA1c ≥ 7% [8].

### 2.6. Laboratory and Clinical Measures

The samples obtained were analyzed at Sembel Hospital. Five mL of blood was obtained from the femoral vein, after 8 hours of fasting. The blood was subsequently aliquoted into appropriate biochemistry tubes for subsequent biochemical analysis. The C-reactive protein (CRP) test kit (Diagnostic Automation/Cortes Diagnostic, Inc.) was used to evaluate CRP. FPG, lipid panel (TG, TC, and HDL-C), and HbA1c were analysed as per manufacturer instruction using Beckman Coulter (AU480 Chemistry System). LDL-C was estimated using the Friedewald formula: LDL = TC − HDL‐C − TG/5 (mg/dL). Patients with TG > 400 mg/dL were excluded in this analysis. Non-HDL was estimated using the following equation: non‐HDL cholesterol = TC–HDL.

American Diabetic Association (ADA) guidelines were used to categorize FPG and HbA1c [8]. Lipid panel components were categorized using the National Cholesterol Education Program-Adult Treatment Panel III (NCEP-ATPIII)—National Cholesterol Education Program-Adult Treatment Panel 3^rd^ level [19]. Dyslipidemia was defined as increased TG, increased LDL-C, and decreased HDL cholesterol.

Blood pressure (BP) was measured as per WHO guidelines using a well-calibrated digital sphygmomanometer (MDF® Lenus Digital Blood Pressure Monitor). Diagnosis of hypertension was based on documented antihypertensive treatment or systolic/diastolic (SBP/DBP) > 140/90 mmHg.

The estimated glomerular filtration rate (eGFR) was calculated by the Modification of Diet in Renal Disease (MDRD) formula: eGFR = 186 × [serum creatinine (mg/dL)]^−1.154^ × (age)^−0.203^ × 0.742 (if female). An eGFR < 60 mL/min/1.73 m^2^ was characterised as reduced eGFR.

## 3. Data Analysis

The data was analysed using Statistical Package for Social Sciences (SPSS) (Version 20.0; IBM, Chicago, IL, U.S.A.). Mean ± 1 standard deviations (SD) and interquartile range (IQR) were computed for specific continuous variables. The Kolmogorov-Smirnov test, Shapiro-Wilk test, and visual inspection of normality plots were used to evaluate Gaussian distribution of data. Relationship between specific independent and dependent variables were tested using the Chi-squared (*χ*^2^) test or Fischer's exact test. In addition to such general-purpose analyses, logistic regression with backward variable removal (backward: conditional) was fitted to identify anthropometric, lifestyle, and clinical parameters which were associated with increased odds of HbA1c > 7%. Subsequently, crude and adjusted odds ratios (OR) and associated 95% confidence intervals (95% CI) were reported. Goodness of fit of the final models was checked by the use of the Hosmer and Lemeshow test. All *p* values were 2-sided, and statistical significance was set at <0.05. *p* values were adjusted using the Bonferroni correction.

## 4. Results

### 4.1. Sociodemographic Characteristics of Study Participants

A total of 320 participants were approached for recruitment in the study. Ultimately, 309 agreed to participate (response rate 96.6%). Altogether, analysis of the data demonstrates that the mean (±SD) age and duration since diagnosis were 57.8 (±11.5) and 12.1 (±7.4) years, respectively. The corresponding mean values for BMI and eGFR were 24.6 (±4.4) and 70.1 (±31.98), respectively. Disaggregation of data with respect to the disparate categorical variables demonstrates that their ages (in years) ranged from 20 to 88 and that the largest age grouping was in the 41–60 years of age range (55.0% of the study participants). Separate analysis indicates that over 70.6% of the study participants had abnormal waist circumference and 44.3% had a BMI > 25 kg/m^2^. The proportion of study participants with reduced eGFR was 36.9%. The corresponding proportion of patients with hypertension was 42.7%. Additional information pertaining to demographic, anthropomorphic, and clinical variables is detailed in Table 1.

### 4.2. Frequency and Factors Associated with Poor Glycemic Control

The main finding of this study was that 76.7% of the study participants had poor glycemic control. The mean (±SD) HbA1c value was 8.6% (±1.36) with a max–min value of 4.6-12%. The data also shows that a significant proportion of participants who were alcohol consumers had poor glycemic control. A large proportion of patients with hypertension also had poor glycemic control. Additional multivariate logistic regression analysis demonstrated that patients with hypertension were more likely to have HbA1c ≥ 7% (AOR = 1.97, 95% CI (1.06–3.56), *p* = 0.021). Likelihood of HbA1c ≥ 7% was also higher in patients with abnormal WHR (AOR = 3.01, 95% CI, 3.01 (1.15–7.92 = 0.024)). Further, a unit reduction in eGFR was associated with HbA1c ≥ 7% (AOR = 0.99, 95% CI (0.98–1 = 0.031)). See Table 2 for additional details.

### 4.3. Frequency of Poor Lipid Control

In order to stratify disparate lipid subfractions into specific risk stratums, we employed the NCEP-ATPIII classification schema. The data is stratified according to sex (see Table 3). The mean (±SD) value for the various lipid parameters for all patients was 47.9 (±11.23) for HDL-C, 133 (±40.56) for LDL-C, 198.4 (±1.48.3) for TG, 217.5 (±49.8) for TC, and 169.6 (±45.7) for non-HDL-C. Overall, 80.9% of the patients were dyslipidemic. It must also be emphasised that none of the patients was on lipid-lowering medication.

### 4.4. Univariate and Multivariable Logistic Regression Analyses of Factors Associated with Poor Glycemic and Lipid Control in DM Patients

Univariate relationship between specific demographic and clinical variables and lipid parameters was evaluated. The logistic regression models of the various lipid analytes and ratios are shown in Table 4.

### 4.5. Total Cholesterol (TC)

In the adjusted multivariate logistic regression model, hypercholesterolemia was significantly related to sex, DM duration, HbA1c, and the presence of hypertension. The likelihood of hypercholesterolemia was significantly higher in females (AOR = 1.7, 95% CI, 1.04-2.73, *p* = 0.035) and lower in patients without hypertension (AOR = 1.9, 95% CI, 1.15-3.16, *p* = 0.016). However, a unit increase in DM duration was associated with a decrease in TC (AOR = 0.96, 95% CI, 0.9-0.99, *p* = 0.004) (Table 5).

### 4.6. Low-Density Lipoprotein Cholesterol (LDL-C)

A multivariate logistic regression model was fitted to identify the factors associated with elevated LDL-C. Accordingly, the abnormal LDL-C level was independently associated with sex—likelier in females (COR = 1.79, 95% CI, 1.5-4.31, *p* = 0.08) (Table 5). In the adjusted model, abnormal TC was associated with being a female (AOR = 2.21, 95% CI, 1.38-3.60, *p* = 0.001) and was less likely in the absence of hypertension (AOR = 0.57, 95% CI, 0.35–0.94, *p* = 0.026) (Table 5).

### 4.7. Triglycerides (TG)

In a separate multivariate analysis, the TG level was associated with sex and waist circumference. Females were less likely to have an abnormal level of TG (AOR = 0.61, 95% CI, 0.37-1.0, *p* = 0.05). In addition, there is a unit increase in waist circumference (AOR = 1.04, 95% CI, 1.0-1.06, *p* = 0.002) and HBA1c (AOR = 1.20, 95% CI, 0.99-1.46, *p* = 0.063) (Table 5).

### 4.8. High-Density Lipoprotein (HDL-C) and Non-HDL-C

In this analysis, a unit increase in WC was associated with increased odds of low HDL-C (AOR = 1.03, 95% CI, 1.01-1.05, *p* = 0.011). In a separate analysis, the data shows that 78% of the study participants had suboptimal non‐HDL > 130 mg/dL. Abnormal non-HDL concentration was significantly associated with duration of disease (AOR = 0.95, 95% CI, 0.86-0.98, *p* = 0.007), increasing HbA1c (AOR = 1.24, 95% CI, 0.98-1.57, *p* = 0.069). In contrast, the absence of hypertension and reducing eGFR were associated with reduced likelihood of suboptimal non-HDL (AOR = 0.41, 95% CI, 0.22-0.77, *p* = 0.005 and AOR = 0.996, 95% CI, 0.992-1.01, *p* = 0.087, respectively) (Table 5).

### 4.9. TG/HDL Ratio

Increased risk of suboptimal TG/HDL was present in 45.3% of the patients. Further, the abnormal TG/HDL ratio was less likely in females (AOR = 0.35, 95% CI, 0.19-0.64, *p* = 0.001) and increasing age (AOR = 0.97, 95% CI, 0.93-0.10, *p* = 0.036). On the contrary, likelihood of TG/HDL‐C ratio ≥ 3 was associated with increasing FPG (AOR = 1.01, 95% CI, 1.00–1.02, *p* = 0.013) and increasing waist circumference (AOR = 1.04, 95% CI, 1.00–1.07, *p* = 0.034) (Table 5).

## 5. Discussion

This study assessed the factors associated with poor glycemic and lipid control in DM patients at a diabetes clinic in Asmara, Eritrea. The main finding was that 76.7% of the respondents had poor glycemic control (HbA1c < 7%). The reported proportions are comparable to those reported in similar studies (cross-sectional in nature) from several countries in SSA—80% in Ethiopia [20], 73.52% in Uganda [14], 69.5% in Kenya [21], and 71.9% in Sudan [22]. In general, and the use of disparate HbA1c cut-offs notwithstanding, these values are patently inferior to those reported in some developed countries: 38% in USA [23] and 43.4% in Korea [24]. Interestingly, previous studies in the same facility conducted in 2009 reported that optimal HbA1c thresholds were achieved by 29.9% of the registered patients [16]. Therefore, it can be argued that there has been a decline in the quality of DM care in this facility. The latter proposition is noteworthy since it emphasises the largely underappreciated fact that sustained maintenance of near-normal or tighter glycemic control in DM patients is a challenge in most countries in SSA.

Multiple studies and reports have concluded that the disproportionate number of DM patients with poor glycemic control is traceable to both institutional and individual factors including drug cost and availability, health policy disparities, limited self-monitoring of blood glucose (SMBG), widespread use of herbal therapies (a problem enhanced by direct-to-consumer advertising), poor prescription patterns by health personnel, and lack of laboratory reagents (HbA1c reagents in particular) [25]. Emphasising some of these factors, a previous research team in the same facility asserted that sustainable upgraded laboratory services combined with training in clinical management may be the key to the attainment of sustainable improvements in DM care in LMIC such as Eritrea [26].

Using an exploratory approach, e.g., lacking preformulated hypotheses, we identified several risk factors which were associated with poor glycemic control. Bivariate analysis demonstrated that poor glycemic control was associated with alcohol consumption, hypertension, WHR, and FPG. Further, the final multivariable model demonstrated that dieting, hypertension, WHR, eGFR, and FPG were associated with poor glycemic control. Needless to say, the link between glycemic control and WHR (marker of abdominal obesity, hence insulin resistance) and high FPG is in line with reports from other studies [27]. Indeed, it is our position that WHR appears to be a better marker of poor glycemic control compared to BMI in this cohort. This finding lends credence to numerous studies which have demonstrated that measures of central adiposity rather than general obesity appear to be better markers of T2DM in populations from SSA [28]. To explain this phenomenon, it has been suggested that age-related increases in total body fat and visceral adiposity compromise the reliability of BMI as a sensitive marker of adiposity in older age groups [29]. Irrespectively, the use of markers of visceral adiposity in SSA is limited by the absence of robust data from prospective, randomized, controlled studies designed to establish locally relevant normal-to-abnormal breakpoints for WC or WHR.

Another notable finding was the proportion of DM patients with hypertension (42.7%) and reduced eGFR (36.9%) (Table 1). Importantly, a recent position statement by ADA reiterated that hypertension is common in DM patients and that it may accentuate cardiovascular risk and contributes disproportionately to the direct and indirect costs of DM [30, 31]. In all, several meta-analyses and systematic reviews have demonstrated that optimal BP management and simultaneous management of concurrent risk factors may reduce risk of ASCVD-related morbidity and mortality [31, 32]. In this regard, optimizing the treatment of DM patients with hypertension is a notable area that requires close consideration. Unfortunately, few countries in SSA have treatment protocols for DM patients and other coexisting diseases (e.g., hypertension, among others).

The independent beneficial impact of nonpharmacologic interventions on glycemic control in DM patients is well documented. Significantly, multiple epidemiologic and trial-level evidence has consistently demonstrated that unhealthy lifestyle habits have a strong influence on glucose-insulin homeostasis and lipid and lipoprotein concentrations [8, 33–35]. Based on these reports, therapeutic lifestyle changes are currently regarded as a cornerstone of DM therapy. Interestingly, the relationship between glycemic control and specific nonpharmacologic interventions evaluated in this study was not significant. Considering the small proportion of participants in this study who were active smokers or alcohol consumers, we can conclude that the study was underpowered to detect such associations. Be that as it may, research has shown that implementing vital lifestyle interventions such as dieting is fraught with multiple challenges. The observed lack of association between poor glycemic control and specific lifestyle intervention measures may in part be explained by suboptimal implementation of these interventions. Needless to say, the shortage of trained medical nutritionists and culturally adapted dietitians is a critical challenge. This problem is also compounded by the fact that nutritional information is lacking for many staple foods in SSA (Eritrea included). Not to be forgotten is the fact that several studies have demonstrated that the efficacy and adherence rates to multimodal behaviour interventions are notoriously poor [36].

Taken together, our analysis has demonstrated that the use of lifestyle interventions in the management of DM patients in this setting is both suboptimal and underutilized in certain respect (only 54% of the participants indicated that they were engaged in some form of exercise). This represents a missed opportunity since nonpharmacologic interventions/self-management support interventions may provide many benefits beyond management of hyperglycemia and lipid levels. For example, they are cost-effective and can be used in the prevention of T2DM and to stem progression from prediabetes to DM or mitigate DM-associated complications [37]. At the very least, additional research on the efficacy of lifestyle interventions in this setting is warranted. In particular, detailed data on the components of lifestyle interventions which are reliably associated with increased effectiveness is required. This information may aide in the development of locally appropriate lifestyle intervention curriculums.

Although DM is generally associated with derangements in glucose metabolism, pathological alterations in lipid metabolism are part of the cluster of cardiometabolic risk factors. The characteristic alterations consist of low HDL-C and high TG [38]. Consistent with previous studies in SSA [39], this study established that a large proportion of the study participants presented with dyslipidemia (80.9%). The use of NCEP-ATPIII risk profiling schema (Table 3) indicates that a large proportion of these patients have antecedent risk of ASCVD and specific microangiopathies [8, 19]. Moreover, the enhanced risk can also be viewed from the fact that a large proportion of the patients presented with hypertension. The high frequency of dyslipidemia in DM patients in SSA can be attributed to multiple factors including low frequency of screening for dyslipidemia, low inclination by health personnel to prescribe antilipid medications, and the costs associated with lipid panel measurements or medications [39].

As we have described above, multivariate analysis demonstrated that the presence of high TC and LDL-C was associated with sex (enhanced risks in females) and presence of hypertension. The comparatively high prevalence of abnormal TC and LDL-C in females has been reported in landmark studies such as the UK Prospective Diabetes Study (UKPDS) [40]. As a whole, the high proportion of patients with elevated LDL-C (81.6% had LDL‐C > 100 mg/dL) should raise concern. The enhanced likelihood of elevated LDL-C in patients with hypertension is also pertinent and exemplifies the urgent need for antilipid medications and/or appropriate nonpharmacologic interventions.

In order to provide a comprehensive assessment of clinical practice in the hospital, non-HDL-C, a surrogate marker of total atherogenic lipoprotein burden and relative risk of ASCVD, was also evaluated. In general, 78% of the study participants did not attain non-HDL-C target (>130 mg/dL). According to the fitted logistic regression model, abnormal non-LDL-C was associated with adverse risk factors such as longer duration of DM, abnormal HbA1c, presence of hypertension, and reduced eGFR in line with previous studies [41]. It has been suggested that in the presence of very high TG levels, non-HDL-C (TC minus HDL-C) will better represent ASCVD risk compared to LDL-C alone [41–43]. It encompasses all cholesterol present in atherogenic lipoprotein particles. Unlike LDL-C, which can incorrectly be calculated using the Friedewald equation in the presence of postprandial TG, non-HDL-C is reliable when measured in the nonfasting state. This point is important because clinics in Eritrea rely on the Friedewald equation to estimate LDL-C. More specifically, some experts have argued that since non-HDL-C can be measured without the requirement for fasting, it can sometimes prove to be cost-effective and beneficial in the clinical practice [44]. At present, specific guidelines (NCEP-ATPIII) also recommend the use of non-HDL-C as a secondary target after correcting LDL-C levels in high-risk individuals [41]. Irrespectively, data on the clinical utility of non-HDL-C for the management of DM patients and related outcomes is lacking in Eritrea. In this regard, the value of local studies cannot be overemphasised.

Up to this point, we have largely discussed the proportions and factors associated with lipid panel abnormalities. At the present time, evidence in the literature suggests that reliance on isolated lipid parameters may miss prognostically relevant lipid abnormalities [44, 45]. Therefore, the several alternatives including the lipid triad concept (high TG, high LDL-C, and low HDL-C)/atherogenic lipoprotein phenotype and lipid ratios (TG/HDL, TC/HDL, and LDL/HDL) have been suggested [44]. Remarkably, our study demonstrated that abnormal TG/HDL profile was associated with adverse risk factors such as high FPG and increasing WC. The connection between TG/HDL, FPG, and WC uncovered in this study is interesting given the fact that abnormal WC has previously been proposed as a marker of insulin resistance [45]. In any case, the highlighted connection can only reinforce our earlier assertion that markers of central adiposity may have greater utility in Eritrea.

## 6. Limitations of the Study

The cross-sectional nature of the study and the dependence on patient-reported medical history are key drawbacks. Miscoding, clerical errors, and unverifiable responses (however marginal) by respondents may also be limiting. The use of self-reported questionnaires was also limiting. To illustrate, lifestyle interventions such as dieting and physical activity were measured using self-reporting questionnaires. It is important to recognize that a major criticism of these self-reports is that they are highly inaccurate and unreliable. In keeping with this caveat, it is difficult to establish whether the affirmative reports of dieting or physical exercising uncovered in the current study met established guidelines. The study may also be hindered by the fact that some vital information was not captured, e.g., adherence to DM medications. Although this facility pools a large percentage of patients from Asmara, it can be argued that the data generated should be used cautiously when making overly broad generalizations on patient care in the city. Irrespectively, it is our belief that the present study provides critical epidemiological information on lipid profiles and the magnitude of poor glycemic control. The patterns of correlating factors highlighted in this study are also pertinent.

## 7. Conclusion

We conclude that a high proportion of diabetes patients in Halibet Regional Referral Hospital have poor glycemic control and dyslipidemia. In addition, the clinical expression of T2DM in this population was characterised by high prevalence of several traits associated with metabolic syndrome (e.g., abnormal WC, dyslipidemia, and hypertension). This outcome implicates inadequate institutional programs or individual factors, e.g., lifestyles and adherence to therapy. Therefore, there is an urgent need for simultaneous improvements in the management of both hyperglycemia and dyslipidemia. This will require evidence-based optimization of pharmacological and nonpharmacological interventions. The data also provides potentially useful insights on hyperglycemia and lipid panel data and correlating demographic, clinical, and laboratory variables. However, the cross-sectional nature of the study implies that the findings of this study are, at the very least, hypothesis-generating. Nevertheless, the study may provide some useful insight for subsequent studies.

## Figures and Tables

**Table 1:** Characteristics of the study population stratified by sex (309).

Variables	Sex		*p* value	Total *N* (%)
	Male *N* (%)	Female *N* (%)		
Patient age (years)				
<40	4 (25.0)	12 (75.0)	0.004	16 (5.2)
41-60	82 (48.2)	88 (51.8)		170 (55.0)
>60	77 (62.6)	46 (37.4)		123 (39.8)
Duration of DM (years)				
<5	29 (50.9)	28 (49.1)	0.815	57 (18.4)
6-10	53 (50.0)	53 (50.0)		106 (34.3)
11-15	26 (51.0)	25 (49.0)		51 (16.5)
16-20	36 (59.0)	25 (41.0)		61 (19.7)
>20	19 (55.9)	15 (44.1)		34 (11.0)
Occupation				
Not employed	28 (18.9)	120 (81.1)	0.001	148 (47.9)
Driver	28 (100)	0 (0)		28 (2.9)
Health worker	2 (22.2)	7 (77.8)		9 (2.9)
Private sector	5 (10.6)	42 (89.4)		47 (15.2)
Public sector	63 (81.8)	14 (18.2)		77 (24.9)
Educational level				
Illiterate	4 (8.5)	43 (91.5)	0.001	47 (15.2)
Primary	57 (50.4)	56 (49.6)		113 (36.6)
Secondary	60 (61.2)	38 (38.8)		98 (31.7)
Tertiary	42 (82.4)	9 (17.6)		51 (16.5)
Dieting (yes)	132 (50.6)	129 (49.9)	0.051	261 (84.5)
Alcohol (yes)	58 (87.9)	8 (12.1)	0.001	66 (21.4)
Physical exercising (yes)	95 (56.9)	72 (43.1)	0.071	167 (54.0)
Smoking (yes)	15 (100)	0 (0.0)	0.001	15 (100)
Hypertension (yes)	63 (47.7)	69 (52.3)	0.293	132 (42.7)
Family member with DM (yes)	60 (54.1)	51 (45.9)	0.411	111 (35.9)
BMI (kg/m^2^)				
<18.5	7 (43.8)	9 (56.3)	0.001	16 (5.2)
18.6–25	96 (61.5)	60 (38.5)		156 (50.5)
25–30	56 (51.9)	52 (48.1)		108 (35.0)
>30	4 (13.8)	25 (86.2)		29 (9.4)
Waist circumference (abnormal)	108 (49.5)	110 (50.5)	0.052	218 (70.6)
eGFR (<60 mL/min/1.73 m^2^)	29 (25.4)	85 (74.6)	0.001	114 (36.9)
CRP (positive)	13 (37.1)	22 (62.9)	0.037	35 (11.3)
FPG (abnormal)	109 (50.2)	108 (49.8)	0.213	217 (70.5)

CRP: C-reactive protein; DM: diabetes mellitus; FPG: fasting plasma glucose; BMI: body mass index; eGFR: estimated glomerular filtration rates.

**Table 2:** Bivariate and multivariable logistic regression analyses of factors associated with poor glycemic control in DM patients at Halibet Regional Referral Hospital, in Asmara, Eritrea (*n* = 309).

Variables	Glycemic control		Crude OR (95% CI)	Adjusted OR (95% CI)
	Good	Poor		
Sex				
Male	39 (23.8)	125 (76.2)	1	
Female	33 (22.8)	112 (77.2)	0.77 (0.35–1.69)	
Age				
<40	3 (18.8)	13 (81.2)	0.92 (0.12-6.73)	
41-60	38 (22.4)	132 (77.6)	0.84 (0.35 -2.02)	
>60	31 (25.2)	92 (74.8)	1	
Duration of DM			**1.05 (0.99–1.11)** ^∗^	
<10 years	42 (25.8)	121 (74.2)		
10-20 years	25 (22.3)	87 (77.7)		
> 20 years	5 (14.7)	29 (85.3)		
Educational level				
Illiterate	12 (25.5)	35 (74.5)	0.18 (0.37-0.92)	
Primary	28 (24.8)	85 (75.2)	0.32 (0.08–1.34)	
Secondary	24 (24.5)	74 (75.5)	0.22 (0.51–0.92)	
Tertiary	8 (15.7)	43 (84.3)	1	
Family member with DM				
No	50 (25.3)	148 (74.7)	1	
Yes	22 (19.8)	89 (80.2)	0.54 (0.24–1.23)	
Dieting				
No	6 (12.5)	42 (87.5)	1	1
Yes	66 (25.3)	195 (74.7)	1.35 (0.40–4.55)	2.4 (0.84–6.86)
Alcohol				
No	**56 (23.0)**	**187 (77.0)**	1	
Yes	**16 (24.2)**	**50 (75.8)**	0.43 (0.13–1.45)	
Exercising				
No	34 (23.9)	108 (76.1)	1	
Yes	38 (22.8)	129 (77.2)	1.01 (0.49–2.11)	
Hypertension				
No	**30 (16.9)**	**147 (83.1)**	**1**	**1**
Yes	**42 (31.8)**	**90 (68.2)**	**1.97 (1.03–3.77)** ^∗^	**1.94 (1.06–3.56)** ^∗^
BMI				
<25	38 (22.1)	134 (77.9)	1	
>25	32 (24.1)	101 (75.9)	0.87 (0.38–1.99)	
Waist/hip ratio				
Normal	**6 (12.0)**	**44 (88.0)**	**1**	**1**
Abnormal	**66 (25.5)**	**193 (74.5)**	**3.00 (1.20–8.23)** ^∗^	**3.01 (1.15–7.92)** ^∗^
eGFR (MDRD) (mL/min/1.73 m^2^)			**0.99 (0.98–1.00)** ^∗^	**0.99 (0.98–1)** ^∗^
>60 mL/min/1.73 m^2^	25 (21.9)	89 (78.1)		
<60 mL/min/1.73 m^2^	47 (24.1)	148 (75.9)		
FPG (mg/dL)			**1.02 (1.01-1.03)** ^∗^	**1.02 (1.01-1.03)** ^∗^
<125 mg/dL	**41 (45.1)**	**50 (54.9)**		
>125 mg/dL	**31 (14.3)**	**186 (85.7)**		

Predicted probability is of membership for poor glycemic control. Good glycemic control was defined as HA1c ≤ 7%. Poor glycemic control was defined as HA1c ≥ 7%. ^∗^Significant association (*p* value < 0.005). BMI: body mass index; DM: diabetes mellitus; eGFR: estimated glomerular filtration rate; MDRD: Modification of Diet in Renal Disease; FPG: fasting plasma glucose.

**Table 3:** Lipid profile of DM patients at Halibet Regional Referral Hospital, in Asmara, Eritrea (*n* = 309).

NCEP-ATPIII classification	Female *N* (%)	Male *N* (%)	*p* value	Frequency *N* (%)
Total cholesterol (mg/dL)				
Optimal serum concentration (<200)	42 (36.8)	72 (63.2)	0.001	117 (36.9)
Borderline (200-239)	45 (45.0)	55 (55.0)		97 (32.4)
High-risk serum conc. (≥240)	59 (62.1)	36 (37.9)		95 (30.7)
Triglyceride (mg/dL)				
Normal (<150)	74 (54.8)	61 (45.2)	0.063	69 (22.3)
Borderline high (150-199)	29 (42.0)	40 (58.0)		105 (34.0)
High (≥200)	43 (41.0)	62 (59.0)		135 (43.7)
LDL-C (mg/dL)				
Optimal (<100)	20 (37.0)	34 (63.0)	0.001	57 (18.4)
Near optimal (100-129)	35 (40.2)	52 (59.8)		84 (27.2)
Borderline high (130-159)	41 (48.8)	43 (51.2)		84 (27.2)
High (160-189)	26 (65.0)	14 (35.0)		40 (13.0)
Very high (≥190)	20 (80.0)	5 (20.0)		25 (8.1)
HDL-C				
Optimal (≥60)	44 (30.1)	13 (8.0)	0.001	57 (18.4)
Borderline men (40-59) and women (50-59)	30 (20.5)	96 (58.9)		126 (40.8)
High risk (<40 men) and (<50 women)	72 (49.3)	54 (33.1)		126 (40.8)

^∗^NCEP-ATPIII: National Cholesterol Education Program-Adult Treatment Panel 3^rd^ level.

**Table 4:** Adjusted logistic regression analysis of factors associated with control of specific lipid analytes in DM patients at Halibet Referal Hospital, in Asmara, Eritrea (*n* = 309).

Variables	TC ≥ 200 mg/dL COR (95% CI)	LDL ≥ 130 mg/dL COR (95% CI)	TG ≥ 150 mg/dL COR (95% CI)	HDL < 40 mg/dL COR (95% CI)	Non‐HDL ≥ 130 mg/dL COR (95% CI)	TG/HDL ≥ 3 COR (95% CI)
Sex						
Male	1	1	1	1	1	1
Female	1.79 (0.96–3.4)	1.79 (0.94–0.34)	4.5 (2.0–10.14)	1.72 (0.79–3.75)	0.97 (0.45-2.08)	0.51 (0.25-1.03)
*p* value	**0.07**	**0.08**	**0.001**	0.09	0.928	0.060
Age of patient	1.01 (0.96-1.03)	1.01 (0.99-1.04)	0.99 (0.96-1.03)	0.99 (0.96-1.02)	1.01 (0.98-1.04)	0.99 (0.96-1.02)
*p* value	0.46	0.39	0.727	0.479	0.47	0.973
Education						
Tertiary	**1**	**1**	1	1	1	1
Illiterate	0.83 (0.31-2.24)	0.86 (0.32-2.4)	0.46 (0.15-1.46)	1.3 (0.47-3.6)	1.3 (0.38-4.5)	0.97 (0.42-3.8)
Primary	0.77 (0.34-1.73)	0.84 (0.40-1.78)	0.55 (0.23-1.33)	1.2 (0.55-2.53)	0.64 (0.30-1.54)	0.85 (0.38-1.9)
High school	0.103 (0.49-2.21)	0.75 (0.35-1.60)	**0.34 (0.23-0.87)** ^∗^	1.08 (0.5-2.33)	0.96 (0.39-2.3)	1.22 (0.55-2.72)
Duration of disease	**0.95 (0.92-0.99)**	0.98 (0.95-1.02)	1.01 (0.97-1.06)	0.996 (0.96-1.04)	**0.94 (0.91-0.98)**	1.01 (0.96-1.06)
*p* value	**0.007**	0.37	0.605	0.846	**0.004**	0.722
eGFR (mL/min/1.73 m^2^)	1.00 (0.99–1.01)	0.99 (0.91–1.0)	1.00 (0.99–1.00)	0.995 (0.99–1.00)	0.99 (0.90-1.001)	1.004 (0.9–1.013)
*p* value	0.981	0.27	0.862	0.074	0.079	0.326
BMI	1.05 (0.96-1.14)	1.08 (1.0-1.18)	1.01 (0.92-1.12)	0.99 (0.92-1.08)	1.19 (0.94-1.45)	1.02 (0.882–1.17)
*p* value	0.29	0.139	0.851	0.892	0.076	0.828
Hypertension						
Present	1	1	1	1	1	1
Not present	0.6 (0.35–1.03)	0.56 (0.35-1.02)	1.74 (0.97-3.15)	0.95 (0.56-1.59)	**1.94 (1.06-3.5)**	1.59 (0.82-3.09)
*p* value	**0.06**	0.059	0.065	0.829	**0.031**	1.69
FPG	1.001 (0.99-1.02)	1.0 (0.99-1.003)	1.00 (0.99-1.01)	0.998 (0.995-1.0)	1.008 (0.998-1.016)	**1.01 (1.00-1.01)**
*p* value	0.652	0.764	0.807	0.19	**0.324**	**0.033**
Physical exercising						
Yes	1	1	1	1	1	1
No	1.07 (0.66-1.75)	1.04 (0.64-1.70)	1.13 (0.64-1.99)	1.03 (0.63-1.69)	1.17 (0.66-2.08)	1.05 (0.56-1.196)
*p* value	0.78	0.787	0.668	0.898	0.596	0.889
Alcohol						
Yes	1	1	1	1	1	1
No	1.06 (0.56-2.00)	1.5 (0.79-3.00)	0.80 (0.36-1.76)	1.09 (0.57-2.12)	1.16 (0.56-2.42)	0.797 (0.35-1.83)
*p* value	0.97	0.201	0.582	0.790	0.690	0.59
Dieting						
No	1	1	1	1	1	1
Yes	0.92 (0.5-1.8)	1.14 (0.57-2.30)	1.15 (0.52-2.56)	0.67 (0.32-1.37)	1.16 (0.46-2.4)	0.82 (0.32-2.12)
*p* value	0.796	0.712	0.728	0.267	0.920	0.683
WC	0.99 (0.96-1.04)	0.96 (0.95-1.02)	1.0 (0.96-1.03)	1.03 (1.0-1.07)	0.598 (0.861-0.995)	**1.04 (1.0-1.10)**
*p* value	0.960	0.39	0.855	**0.058**	0.598	**0.0231**

BMI: body mass index; eGFR: estimated glomerular infiltration rate; FPG: fasting plasma glucose; LDL: low-density lipoproteins; TG: triacylglycerol; HDL: high-density lipoproteins; WC: waist circumference. Bold values or ∗: *p* value < 0.005.

**Table 5:** Multivariate logistic regression analysis of factors associated with control of specific lipid subfractions in DM patients at Halibet Regional Referral Hospital, in Asmara, Eritrea (*n* = 309).

Variables	TC ≥ 200 mg/dL AOR (95% CI)	LDL ≥ 130 mg/dL AOR (95% CI)	TG ≥ 150 mg/dL AOR (95% CI)	HDL < 40 mg/dL men and <50 women AOR (95% CI)	Non‐HDL ≥ 130 mg/dL AOR (95% CI)	TG/HDL ≥ 3 AOR (95% CI)
Sex						
Male	1	1	1	1		1
Female	1.7 (1.04-2.73)	2.21 (1.38-3.6)	0.56 (0.35-0.88)	1.9 (1.08–3.39)		0.35 (0.19–0.64)
*p* value	**0.035**	**0.001**	**0.006**	**0.026**		**0.001**
Age of patient						0.97 (0.93-0.10)
*p* value						**0.036**
Duration of disease	0.96 (0.9-0.99)				0.95 (0.86-0.98)	
*p* value	**0.004**				**0.007**	
Hypertension						
Present	1	1			1	
Not present	0.54 (0.33–0.89)	0.57 (0.35-0.94)			0.41 (0.22-0.77)	
*p* value	**0.016**	**0.26**			**0.005**	
eGFR (mL/min/1.73 m^2^)					**0.996 (0.992–1.01)**	
*p* value					**0.087**	
FPG						1.01 (1.00-1.012)
*p* value						**0.013**
HbA1c	1.2 (0.98–1.47)		1.20 (0.99–1.46)		1.24 (0.98–1.57)	
*p* value	0.73		0.063		0.069	
WC			1.04 (1.0-1.06)	1.03 (1.01-1.05)		1.04 (1.0-1.07)
*p* value			**0.002**	**0.011**		**0.034**

BMI: body mass index; eGFR: estimated glomerular infiltration rate; FPG: fasting plasma glucose; LDL: low-density lipoproteins; TG: triacylglycerol; HDL: high-density lipoproteins; WC: waist circumference. Bold letters or ∗: *p* value < 0.005.

## Data Availability

The data used to support the findings of this study are available from the corresponding author upon request.
